# Towards a structural biology of the hydrophobic effect in protein folding

**DOI:** 10.1038/srep28285

**Published:** 2016-07-27

**Authors:** Carlo Camilloni, Daniela Bonetti, Angela Morrone, Rajanish Giri, Christopher M. Dobson, Maurizio Brunori, Stefano Gianni, Michele Vendruscolo

**Affiliations:** 1 Department of Chemistry, University of Cambridge, Cambridge CB2 1EW, UK; 2 Istituto Pasteur – Fondazione Cenci Bolognetti and Istituto di Biologia e Patologia Molecolari del CNR, Dipartimento di Scienze Biochimiche “A. Rossi Fanelli” Università di Roma “La Sapienza”, 00185 Rome, Italy

## Abstract

The hydrophobic effect is a major driving force in protein folding. A complete understanding of this effect requires the description of the conformational states of water and protein molecules at different temperatures. Towards this goal, we characterise the cold and hot denatured states of a protein by modelling NMR chemical shifts using restrained molecular dynamics simulations. A detailed analysis of the resulting structures reveals that water molecules in the bulk and at the protein interface form on average the same number of hydrogen bonds. Thus, even if proteins are ‘large’ particles (in terms of the hydrophobic effect, i.e. larger than 1 nm), because of the presence of complex surface patterns of polar and non-polar residues their behaviour can be compared to that of ‘small’ particles (i.e. smaller than 1 nm). We thus find that the hot denatured state is more compact and richer in secondary structure than the cold denatured state, since water at lower temperatures can form more hydrogen bonds than at high temperatures. Then, using Φ-value analysis we show that the structural differences between the hot and cold denatured states result in two alternative folding mechanisms. These findings thus illustrate how the analysis of water-protein hydrogen bonds can reveal the molecular origins of protein behaviours associated with the hydrophobic effect.

A detailed understanding of the molecular origins of the hydrophobic effect^1^,^2^,^3^,^4^,^5^,^6^,^7^,^8^,^9^,^10^ in proteins and of its role as a driving force in protein folding and assembly^10^,^11^,^12^,^13^,^14^ is still an open problem. This situation arises because protein sequences include a complex combination of hydrophobic (non-polar) and hydrophilic (polar) regions. The resulting patterns of non-polar regions have typical dimensions that correspond to a critical value for the hydrophobic effect^1^,^2^,^3^,^4^,^5^,^6^,^7^,^8^. This value, which is of about 1 nm, corresponds to the distinction between small and large non-polar solutes^1^,^2^,^3^,^4^,^5^,^6^,^7^,^8^,^9^. To understand the molecular origin of this characteristic length, one should start from the observation that water molecules tend to form networks of hydrogen bonds at the expense of their rotational entropy. Non-polar solutes smaller than 1 nm occupy a volume that is too small to perturb significantly the formation of such hydrogen bond networks. By contrast water molecules near the surfaces of non-polar solutes larger than 1 nm cannot form all the hydrogen bonds that they would do in bulk. These two situations correspond to a different scaling of the hydrophobic forces, with the volume or the surface, for small or large ideal hydrophobic solutes, respectively^1^.

Here our aim is to clarify first whether proteins, whose surfaces as mentioned above are characterized by the presence of complex polar and non-polar patterns, behave effectively like small or large non-polar solutes, and then to investigate the consequences of this fact on their folding behaviour^11^,^12^,^13^. Addressing this question requires an accurate characterization of the conformational space of a protein in water to study the number of hydrogen bonds formed by water molecules in proximity of its surface with respect to the bulk.

To attack this problem, we exploited the opportunities offered by the case of yeast frataxin (Fig. 1a). Frataxin, which is a protein involved in the assembly of iron-sulfur clusters, is also related to Friedreich’s ataxia, a fatal neurodegenerative condition^15^. We considered yeast frataxin because this protein represents one of the few examples for which both cold and hot denatured states have been observed at neutral pH and without addition of destabilizing agents (at 272 K and 323 K, respectively), and characterized by nuclear magnetic resonance (NMR) chemical shifts and circular dichroism (CD)^15^,^16^,^17^,^18^. As shown by the pioneering work of Privalov, the thermodynamic analysis of cold denaturation, compared to that of the thermal denaturation, offers unique advantages for understanding the molecular determinants of hydrophobicity^19^,^20^. Indeed, it is generally recognised that while thermal denaturation is the consequence of a temperature-induced increase in conformational fluctuations, cold denaturation is a consequence of an enthalpy gain of the solvent^19^,^20^. Not many atomistic details are known, however, about the structural consequences of this gain of entropy or enthalpy in the hot and cold denatured states, respectively. Thus, modelling the cold and hot denatured states of yeast frataxin in the absence of any additional agent should highlight the different roles played by the protein and solvent at different temperatures.

In the following we employ the chemical shifts measured in cold and hot denaturation conditions^15^,^16^,^17^ together with replica-averaged metadynamics (RAM) simulations^21^,^22^ (see Materials and Methods) to elucidate at atomic resolution the structure and the dynamics of the cold denatured state (CDS) and hot denatured state (HDS). In the RAM simulations the experimental information provided by NMR chemical shifts is incorporated in terms of structural restraints^22^ and at the same time the sampling of the conformational space is enhanced by metadynamics^23^. This overall approach makes it possible to simultaneously change the force field used in the molecular dynamics simulations to improve their agreement with the experimental data in the spirit of the maximum entropy principle^24^ and to significantly decrease the computational resources required to obtain convergence in the sampling. Then, by using Φ-values analysis^25^,^26^,^27^ and Φ-values restrained molecular simulations^26^, we determined their cold transition state (CTS) and hot transition state (HTS) to describe the differences in the cold and hot denaturation processes corresponding to the differences between the cold and hot denatured states.

## Results and Discussion

### Determination of the hot and cold denatured state ensembles

**Figure 1:**
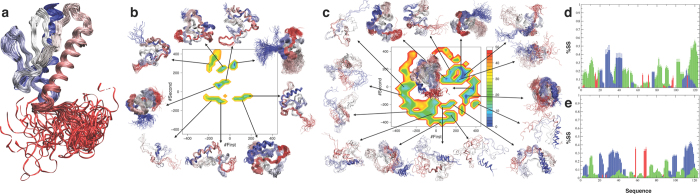
The cold and hot denatured states of frataxin are structurally different. (**a**) Structure of the native state of frataxin. (**b**) Free energy surface of the cold denatured state (CDS) determined at 272 K as a function of the sketch-map^63^ collective variables that describe the conformational features of the ensembles (see Methods). Nine microstates are shown representing the local and global minima. These microstates comprise >90% of the total population. (**c**) Free energy surface of the hot denatured state (HDS) determined at 323 K as a function of the same two collective variables. Sixteen microstates are shown representing the local and global minima. These microstates comprise >90% of the total population (see Methods). (**d,e**) Secondary structure populations of the CDS (**d**) and HDS (**e**). α-Helices are represented in blue, β-strands in red and polyproline II in green; free energies are given in kJ/mol.

From the analysis of the RAM simulations at convergence (Fig. S1) we conclude that the two denatured states exhibit qualitatively different behaviours, as the regions of conformational space that they sample are essentially non-overlapping (Fig. 1b,c). None of the most populated microstates in the CDS is equally populated in the HDS and vice versa. Furthermore, the conformational space of the CDS is significantly smaller than that of HDS, with less local minima being populated in the CDS than in the HDS (Fig. 1b,c). In the CDS, 9 minima cover the 90% of the conformational space while 16 much broader minima are needed in the HDS to cover the same extent. Structurally the CDS is more expanded than the HDS. Furthermore, the α-helical content is 6% in the CDS and 10% in the HDS (Fig. 1d), and the β-sheet content is 0.7% in the CDS and 1.4% in the HTS (Fig. 1e). In the CDS there are three short partially populated α-helices in the region of residues 20–45 and a longer, less populated one, in the region of residues 107–120 in agreement with a previous observation about the presence of residual α-helical structure at the N-terminus^16^. In the HDS instead, there is a larger α-helical population distributed over a broader range of residues with two α-helices in the regions of residues 25–48 and 110–120. A remarkable difference between the two ensembles is also observed in the polyproline II content, which is 15% in the CDS and 5% in the HDS. The two ensembles are also different in terms of their radii of gyration (R_g_), with 1.7 nm in the CDS and 1.6 nm in the HDS; for reference, this value is 1.5 nm in the native state (NS), even if this difference is may be underestimated because of known limitations of current force fields^28^. The higher secondary structure content and the lower R_g_ of the HDS correspond to a slightly higher resemblance of the NS to the HDS with respect to the CDS. The average fraction of native contacts^29^, Q, is 0.18 and 0.22 for the CDS and the HDS, respectively (Fig. S2). Overall, the HDS is richer in secondary structure and more compact than the CDS. These observations agree and extend previous comparisons of the two denatured states based on single-molecule FRET, NMR and SAXS measurements^16^,^30^.

### Structural characterization of the hydrophobic effect in protein folding

**Figure 2:**
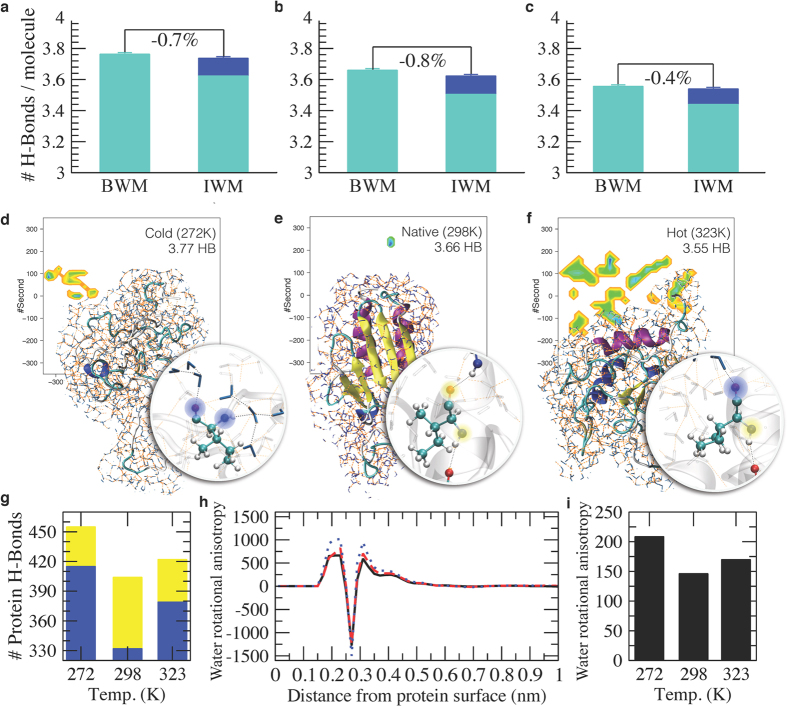
Structural characterization of the hydrophobic effect in protein folding. (**a–c**) Number of hydrogen bonds per water molecule for the CDS (272 K), NS (298 K) and HDS (323 K). The number of hydrogen bonds is calculated for bulk water molecules (BWM, farther than 0.4 nm), and for interface water molecules (IWM, water molecules in the first shell around the protein, within 0.4 nm) considering both the water-water hydrogen bonds (light blue) and the water-protein hydrogen bonds (blue). In our calculations, we found that bulk and interface water molecules form the same number of hydrogen bonds at the three temperatures. (**d–f**) At cold denaturation temperatures water molecules win the competition with the protein to form hydrogen bonds with the protein hydrogen bond donors and acceptors. Two hydrogen bonds formed by a hydrophobic residue are shown in particular, for which only the backbone amide and carbonyl groups can form hydrogen bonds; these two water-protein hydrogen bonds are highlighted in blue. By contrast, under folding conditions intra-protein hydrogen bonds are preferentially formed, and no particular preference is present at hot denaturation temperatures; the two protein-protein hydrogen bonds (within a α-helix) are highlighted in yellow. (**g**) Number of hydrogen bonds formed by frataxin in the CDS, NS and HDS ensembles. The number of protein-protein hydrogen bonds is shown in yellow, and that of protein-water hydrogen bonds in blue. (**h**) Water rotational anisotropy as a function of the distance from the protein surface for the CDS (dotted blue), NS (solid black) and HDS (dashed red), calculated as <3cos^2^ϑ(r) −1 > RDF(r), where ϑ is the angle of the vector from a solvent oxygen to a protein atom with the normal of the solvent plane averaged over all the solvent molecules and all the protein atoms, and RDF(r) is the non-normalised radial distribution function of the water with respect to the protein surface. (**i**) Total water rotational anisotropy in presence of the protein calculated as the integral of the absolute value of the former curves. The degree of order of the water molecules near the protein correspond to the extent to which they form hydrogen bonds with the protein, which is large at cold denaturing conditions, small under folding conditions, and intermediate at hot denaturing conditions.

**Figure 3:**
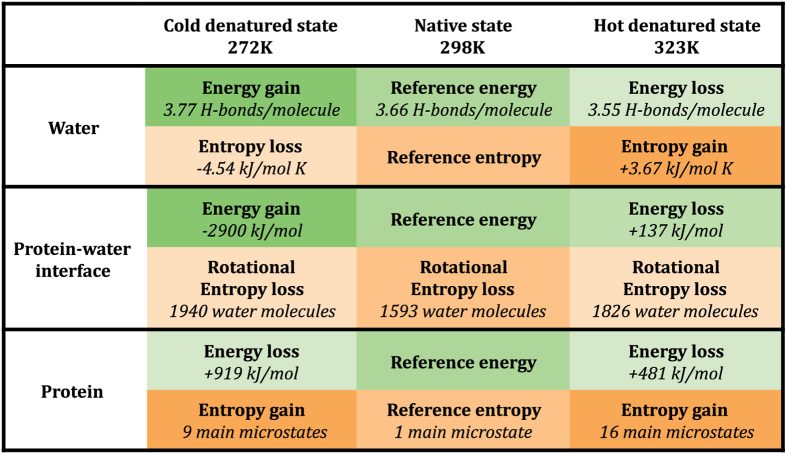
Summary of the entropic and energetic contributions to the cold and hot denatured states.

The different structures of the CDS, NS and HDS correspond to differences in the overall properties of the protein-water system (Fig. 2). To investigate these differences, we first calculated the number of hydrogen bonds in the three states. In increasing the temperature, bulk water molecules (BWM) form decreasing numbers of hydrogen bonds^31^,^32^,^33^, in the current case by going from 3.77 at 272 K, to 3.66 at 298 K, to 3.55 at 323 K (Figs 2a–c and 3 and Fig. S3). Remarkably, these numbers are rather well preserved also for water molecules in the first shell around the protein (interface water molecules, IWM, Fig. 2a–c). In this interface region the number of water-water hydrogen bonds per molecule drops (Fig. 2a–c, light blue), but the total number of hydrogen bonds per molecule is recovered within less than 1% when one takes into account also the protein-water hydrogen bonds (Fig. 2a–c, blue). In essence, while the overall number of hydrogen bonds formed by a water molecule is relatively constant for water molecules in the bulk and at the interface^10^, the protein itself respond to the change in this number with temperature by redistributing its protein-protein (Fig. 2d–f, yellow) and protein-water (Fig. 2d–f, blue) hydrogen bonds, for example by populating polyproline II conformations in the CDS in place of α-helical conformations in the HDS (Fig. 1, in particular in the C-terminal α-helix). In decreasing the temperature from 298 K to 272 K the interface water molecules in the CDS should ideally gain a total of about 94 hydrogen bonds to raise their average value per molecule from 3.66 to 3.77. Our results indicate that water forms 83 such hydrogen bonds with the protein, thus stabilising its CDS (Fig. 2g).

We obtained additional insight into the factors that determine the cold and hot denaturation processes by calculating the van der Waals and the Coulomb energies of the protein-water system (Fig. S4). While the protein-protein energy is minimized in the folded state, the protein-water energy is strengthened under cold denaturation conditions, and is slightly weakened in hot denaturation conditions with respect to the NS. These results suggest that the CDS is stabilized by interactions with the solvent and as a consequence the protein is more expanded. In the NS the balance of the interactions is moved towards the protein-protein interactions and indeed the protein is structured. Finally the HDS exhibits an intermediate behaviour where the protein conserves some residual structure and remains more compact.

A qualitative analysis of the role entropy is obtained by analysing the average orientations of water molecules with respect to the protein as a function of their distances from the protein surface in the CDS, the HDS and the NS (Fig. 2h). A value of zero indicates that on average a water molecule at a given distance does not have any preferred orientation with respect to the protein and so its rotational entropy is the same as in bulk water. Positive and negative values report about an anisotropic distribution of the solvent with respect to the protein and correspond to an entropy loss with respect to the bulk; these curves take also into account the number of water molecules at a given distance (Fig. 2). Up to 0.7 nm from the protein water molecules show an anisotropic distribution with respect to the protein, and a consequent entropy loss with respect to the bulk. This distance corresponds to the first two shells of water around the protein (Fig. S5). In the denatured states this behaviour is slightly more pronounced than in the NS, due to the more exposed surfaces and as a consequence the larger number of water molecules that are oriented. The integral of the absolute value of the curves of Fig. 2h reports on the overall entropy loss with respect to bulk water (Fig. 2i). The NS shows the least water entropy loss, indicating that in the native state the hydrophobic forces, here interpreted essentially in terms of loss of rotational freedom, are minimized.

The structural analysis described above characterizes the entropic and energetic contributions to the folding process of frataxin as a function of the temperature (Fig. 3). In decreasing the temperature from 298 K to 272 K, water gains in energy by increasing by about 3% the number hydrogen bonds, and correspondingly loses in entropy because of the increase in order. At the same time, the protein unfolds by gaining energy by interacting more strongly with the solvent at the expense of protein-protein interactions (Fig. 2b,c), and gaining some entropy by accessing a slightly larger conformational space (Fig. 1a). Water molecules in the first two shells around the protein lose in entropy with respect to the bulk (−12%). By contrast, in increasing the temperature from 298 K to 323 K water loses energy by decreasing by about 3% the number hydrogen bonds and gains entropy because of the decrease in order. In parallel, the protein unfolds, losing energy both due to loss of protein-protein and protein-water interactions and gaining entropy by accessing a larger conformational space (Fig. 1b,c). Water molecules in the first two shells around the protein lose in entropy with respect to the bulk (−11%).

It is also remarkable that both at low and at high temperatures the number of hydrogen bonds per surface water molecule does not depend on the free energy of the conformation in the ensemble (Fig. S6), although the number of hydrogen bonds depend on the identity of the amino acid and to the degree of solvent exposure (Figs S7–S9). This result is particularly important because it shows that the number of number of hydrogen bonds per surface water molecule is a property robust with respect to possible inaccuracies in the simulations, as for example a slightly overestimated compactness of the protein. While these results are consistent with previous observations about the relevance of protein-water interactions in driving cold denaturation^9^,^10^,^16^,^34^,^35^, here we show explicitly that the number of hydrogen bonds is the same for bulk and interface water molecules at all the relevant temperatures. This phenomenon could determine the properties of the folded and unfolded states, as also recently suggested using a two-dimensional model^36^.

### Frataxin folds following different pathways from the cold and hot denatured states

The differences observed for the residual structure of the two denatured state ensembles together with the differences observed in the protein-water interactions offer the opportunity to investigate the consequences of the denaturing conditions on the protein folding process. To this end, we carried out a Φ-value analysis^25^,^27^ in combination with restrained molecular dynamics simulations^26^ to determine the structures of the cold and hot transition states (CTS and the HTS, see Supporting Information). Here Φ values are structurally interpreted as the fraction of native contacts formed by a residue in the transition state and these fractions are thus used as restraints in a simulation to find an ensemble of conformations in agreement with all the Φ values^26^. The Φ values (Table S1 and Fig. S10) show that the hot and cold denaturation pathways are characterized by two structurally different transition states, which provide a structural perspective on the folding reaction.

**Figure 4:**
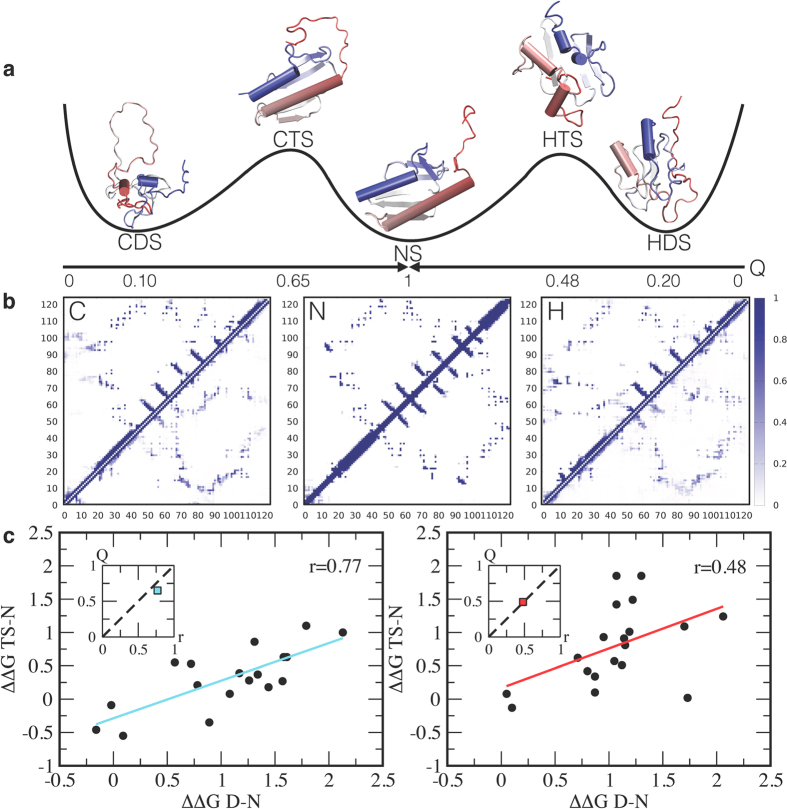
Frataxin folds following different pathways from the cold and hot denatured states. (**a**) Schematic representation of the cold and hot folding pathways along the fraction of native contacts Q. The structural ensembles shown correspond the most populated microstates in the two denatured states, the two transition states and the native state. (**b**) Contact maps for the cold denatured, native and hot denatured states; the color code corresponds to the fraction of contact formation. The contact map of the cold transition state (upper half) and the cold denatured states (lower half) is shown on the left, the contact map for the native state in the middle, and the contact map of the hot transition state (upper half) and the hot denatured states (lower half) on the right. (**c**) Brønsted plots determined at 287 K and 323 K from mutagenesis (see Methods). At low temperature the data are more linearly correlated than at high temperature, corresponding to a higher degree of structure in the cold transition state than in the hot transition state, as reported by the corresponding values of Q (insets).

A comparison of the contact maps of the CTS and HTS and those of the CDS, HDS and NS (Fig. 4a,b) reveals that in the CTS almost all the native secondary structures are already in place, whereas the HTS is more disordered. Also the tertiary contacts are more native-like in the CTS than in the HTS, in particular in the regions corresponding to the docking of the α-helices with the six-stranded β-sheet, as observed in urea^17^. Also, the fraction of native contacts Q is 0.65 and 0.49 for the CTS and HTS, respectively (see also Fig. S2).

A comparison between the structures of CDS, HDS, CTS and HTS allows extracting a structural depiction of the so-called anti-Hammond effect, which is sometimes observed in protein folding^37^. The anti-Hammond effect occurs when the transition state moves towards the product as the reactant becomes more stable. In case of frataxin, while the CDS is less structured than the HDS, the CTS and HTS display opposite behaviours, with the HTS being less structured than the CTS. To provide a structural rationalisation of this behaviour, it may be considered that, because the CDS is very heterogeneous, the free energy well associated with it is shallower than that of the HDS as a function of Q (Fig. S11). Thus, the intersection between the free energy wells of the native and denatured states, which identifies the transition state of the reaction, may shift towards the native conformation, resulting in an apparent anti-Hammond effect.

The solution structures of the CDS, HDS, CTS and HTS reveal important features of the cold and hot folding mechanisms. The increase of residual structure in the denatured state from the CDS to the HTS shifts the folding mechanisms from nucleation-condensation^38^ (where the protein folds as a single cooperative unit) to diffusion-collision^39^ (where the protein folds in contiguous blocks). A signature of these different behaviours lies in the so called Brønsted plot^37^, which relates the activation free energy of a reaction with the corresponding equilibrium free energy and is linear in the nucleation-condensation scenario, while is scattered in diffusion collision^37^. The analysis of the Brønsted plot of the CTS and HTS (Fig. 4c) provides a direct test of the effect of the residual structure in the denatured states on the folding mechanisms. At low temperature, with a weakly structured CDS, there is a strong correlation (0.77) between ΔΔG_D-N_ and ΔΔG_TS-N_, while at high temperature the correlation is weaker (0.48) (Fig. 4c). These results provide a demonstration of the correlation between the fraction of native contacts in the transition state, Q, and the coefficient of correlation between ΔΔG_D-N_ and ΔΔG_TS-N_ (Fig. 4c, insets), and reveal its links with the hydrogen bonding behaviour of water at low and high temperatures and thus with the hydrophobic effect.

## Conclusions

The results that we have presented provide an atomistic representation of the seminal observations by Privalov about the favourable hydration of protein upon cold denaturation^19^,^20^. We have shown how a major role in determining the state of yeast frataxin at different temperatures is played by the competition between water and protein molecules for forming hydrogen bonds with the protein hydrogen bonding donors and acceptors. Our results indicate that this protein interacts differently with water molecules at low and high temperatures, resulting in a more expanded and less structured denatured state at low temperatures, and in a more compact and structured denatured state at high temperatures.

These differences also determine the specific changes in the cold and hot folding pathways of this protein, and they suggest the possible origins of complex effects such as the anti-Hammond behaviour and the Brønsted plot in protein folding by linking them with the number of hydrogen bonds that water molecules tend to form. Upon increasing the temperature from cold to native conditions, as the number of protein-water hydrogen bonds decreases, the protein can form stable secondary and tertiary structures. Then, when the temperature is raised from native to hot denaturing conditions, thermal fluctuations destabilise the native fold, but at the same time a further decrease in the number of protein-water hydrogen bonds results in the conservation of many of secondary structure elements and in a relatively compact denatured state.

These observations may also explain the finding of an increase in compactness and secondary structure content for denatured and for intrinsically disordered proteins upon increasing temperatures^30^,^40^,^41^. Since intrinsically disordered proteins are richer in polar groups than globular proteins, by increasing the temperature, the loss of protein-water hydrogen bonds is rescued by protein-protein hydrogen bonds resulting in more compact conformations than those observed by standard proteins of similar length^30^,^40^,^41^.

**Figure 5:**
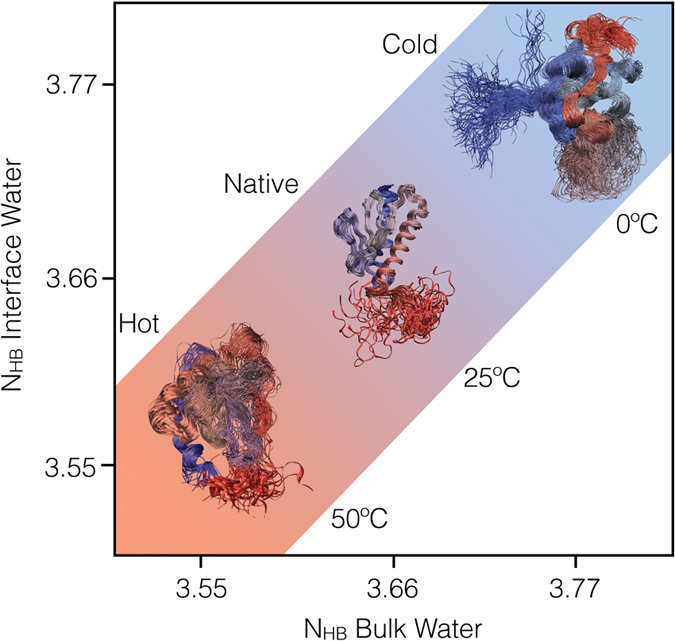
Our calculations indicate that bulk and surface water molecules form the same number of hydrogen bonds. As a consequence of this result, as the temperature is varied the conformation of the protein should adapt to this requirement which causes it to unfold both a low and at high temperatures.

In summary, we have shown that the analysis of the hydrogen bonds between water and protein molecules can provide a detailed characterisation of the hydrophobic effect in protein folding. Our results suggest that proteins behave rather closely according to the classic description of small hydrophobic particles^1^,^42^,^43^,^44^,^45^, as we show that interface water molecules do not lose hydrogen bonds with respect to bulk water (Fig. 5), but only rotational entropy.

## Materials and Methods

### Molecular dynamics simulations

Molecular dynamics simulations of yeast frataxin were performed using the Amber03W force field^46^ with the TIP4P05 water model^47^. All the simulations were run in GROMACS^48^ using PLUMED2^49^ and Almost^50^. A time step of 2 fs was used together with LINCS constraints^51^. Van der Waals and Coulomb interactions were implemented with a cutoff at 0.9 nm, and long-range electrostatic effects were treated with the particle mesh Ewald method^52^ on a grid with a mesh of 0.1 nm. All simulations were carried out in the canonical ensemble at constant volume and by thermosetting the system using a stochastic velocity rescaling^53^. The starting conformation was taken from an available NMR structure (PDB code 2GA5) and solvated with 19,000 water molecules and 15 sodium ions. A standard 100 ns molecular dynamics simulation at 298 K was performed as a reference for the native state ensemble.

### Replica averaged metadynamics (RAM) simulations of the cold and hot denatured state ensembles

A high-temperature (450 K) 30 ns preliminary unfolding simulation was used to select four starting conformations. Each conformation was then subsequently relaxed at the two target temperatures (272 and 323 K) for 10 ns at constant pressure. The final box size was adjusted to a common average value at each temperature and the systems have been additionally relaxed for 10 ns at constant volume. RAM simulations^21^,^22^ were performed using chemical shifts (BMRB depositions 17068 and 17641) as replica-averaged restraints^21^,^22^ and bias-exchange metadynamics^54^ following a protocol recently introduced to sample the denatured state of ACBP^22^. Briefly, four replicas of the system were simulated in parallel at the target temperature with a restraint applied on the average value of the CamShift^55^,^56^ back-calculated NMR chemical shifts^56^ with a force constant set to 24 kJ/(mol ppm^2^). In this way the system evolves with a force field that is perturbed in such a way to increase the agreement with the experimental chemical shifts as resulting by the application of the maximum entropy principle^24^,^57^,^58^,^59^. In principle the number of replicas can be increased at expense of an increasing computational cost, but, as previously shown^21^, four replicas are sufficient to recover with very good accuracy the dynamics from chemical shifts. Four collective variables (CVs) have been employed to enhance the search in the conformational space with bias-exchange metadynamics: the total α-helical content^48^, the total β-sheet content^48^, the radius of gyration and the number of contacts between the regions containing the native α-helices and the native β-sheet. The choice of the secondary structures and radius of gyration as CVs was guided by the hypotheses that transient secondary structures formation and the volume fluctuations of the polypeptide chain capture to a good extent the relevant dynamics of a disordered protein. The additional choice of the number of contacts between the regions containing the native helices and the native β-sheet was suggested by the fact that in a recent characterization of the transition state ensembles frataxin folding from an urea-denatured state most of the lost contact in the transition state are in this region. Gaussians deposition was performed with an initial rate of 0.125 kJ/mol/ps, where the σ values were set to 0.16, 0.1, 0.02 and 0.17, for the four CVs, respectively. In order to keep under control the convergence of the simulations we rescaled the height of the Gaussians using the well-tempered scheme^60^ with a bias-factor of 10. Furthermore, in order to limit the extent of accessible space along each collective variable and correctly treat the problem of the borders, we set the bias as constant outside a defined interval for each CV^61^, as it has been shown that this approach lead to a correct reconstruction of a one-dimensional free energy landscape inside the chosen range; intervals were set to 0–14, 0–10, 1.2–4.0 and 1–18 for the four CVs, respectively. Each replica have been evolved for 800 ns, with exchange trials every 50 ps. Protein conformations have been saved every 20 ps and full systems conformations (protein and solvent) have been saved every 100 ps for further analysis.

### Cold and hot transition states ensembles

Cold and hot transition state ensembles were determined following a standard procedure based on the interpretation of Φ-value analysis in terms of fraction of native contacts^26^. Briefly, given a set of experimental Φ−values, a pseudo energy term has been added to the force field as the squared difference between experimental and simulated Φ−values in order to maximize the agreement with the experimental value while keeping the simulation stable. The Φ−value for a residue *i* is calculated from the fraction of native contacts that it makes in a conformation. Given two residues that are not nearest neighbours, the native contacts between them are defined as the number of heavy side-chain atoms within 0.65 nm in the native structure. With this approach only Φ−values between 0 and 1 can be incorporated as structural restraints.

The different transition state ensembles were generated using simulated annealing. Each ensemble is the results of 300 annealing cycles, 150 ps long, in which the temperature is varied between 272 K or 323 K and 500 K. Only the structures sampled at the reference temperatures are retained for further analysis, resulting in TSE of ~1000 structures each.

### Bulk water simulations

A parallel tempering simulation^49^ of a box of 714 TIP4P05 water molecules was performed to analyse the property of water using seven replicas at 272, 274, 283, 292, 302, 312 and 323K, respectively. Each replica has been evolved for 20 ns in the NPT ensemble using the Parrinello-Rahman barostat^62^ and try exchanges every 0.5 ps.

## Additional Information

**How to cite this article**: Camilloni, C. *et al*. Towards a structural biology of the hydrophobic effect in protein folding. *Sci. Rep.* **6**, 28285; doi: 10.1038/srep28285 (2016).

## Supplementary Material

Supplementary Information
