# Supplementary material for: Towards a structural biology of the hydrophobic effect in protein folding
Source: Sci Rep. 2016 Jul 27;6:28285. doi: 10.1038/srep28285 (PMC4962056; doi:10.1038/srep28285)
Supplement: Supplementary Information [file srep28285-s1.pdf]

SUPPORTING INFORMATION

Towards a structural biology of the hydrophobic effect in protein folding

Carlo Camilloni[1], Daniela Bonetti[2], Angela Morrone[2], Rajanish Giri[2],
Christopher M. Dobson[1], Maurizio Brunori[2], Stefano Gianni[2] and Michele Vendruscolo[1]

[1]*Department of Chemistry, University of Cambridge, Cambridge CB2 1EW, UK*
[2]*Istituto Pasteur – Fondazione Cenci Bolognetti and Istituto di Biologia e Patologia Molecolari del CNR, Dipartimento di Scienze Biochimiche "A. Rossi Fanelli" Università di Roma "La Sapienza", 00185 Rome, Italy.*

Analysis of the denatured state ensembles. The sampling of the four replicas was combined using a standard weighted histogram analysis[1] to generate a four-dimensional free energy landscape where a set of microstates is identified by dividing the four-dimensional CV-space into a homogeneous grid of small dimensional hypercubes. A more general structural description of the ensembles, independent from the initial choice of the CVs, was obtained using the recently developed SketchMap projection technique[2]. Sketchmap provides a way of reducing a multidimensional representation to two dimensions by means of Mercator–like approach in which the distance between two structures in the multidimensional space is qualitatively preserved after a rescaling through a step function. In the present case we analysed both the CDS and HDS at the same in terms of the 256 backbone dihedral angles of Frataxin and we employed SketchMap to identify two parameters (or CVs), common to the two ensembles, for the projection of the free energy. We then used these two CVs together with the four old ones to build a new bi-dimensional free energy landscape as a function of the SketchMap CVs only (Figure 1a,b).

Φ value analysis. The Φ value analysis relies on measuring the effect of mutations on the folding of a given protein[3,4]. By systematically mutating residues, while probing the effect of

the mutation on the folding kinetics and native state stability, it is possible to map one by one interaction patterns in the transition states. The relative formation of the contact is commonly called the Φ value and, as detailed below, can be also used as a restrain for molecular dynamics simulations to determine the structures of the transition state ensembles at nearly atomic resolution[5].

Because our aim was to compare the hot and cold denaturation pathways of frataxin, we analyzed the folding equilibrium and kinetics of frataxin as a function of temperature, and then compared it with different site-directed mutants (Table S1 and Figure S3). All the site directed variants, as well as wild-type frataxin, were subjected to thermal induced denaturation equilibria, monitored by CD spectroscopy, and temperature jump relaxation kinetics, in analogy to what recently reported[6].

The analysis of the kinetic folding mechanism of frataxin as a function of temperature requires a deconvolution of the folding and unfolding components from the observed rate constants. Thus, given that the observed rate constant is governed by the sum of the rate constants for the forward and reverse reactions, we calculated the folding (k_F) and unfolding (k_U) rate constants by using the thermodynamic parameters obtained from equilibrium thermal denaturation experiments (see above). The following equations were employed

$$K_{D-N}(T) = \frac{k_F(T)}{k_U(T)} \qquad \text{(Eq. S1)}$$

$$k_{obs} = k_F(T) + k_U(T) \qquad \text{(Eq. S2)}$$

where $K_{D\text{-}N}$ represents the equilibrium constant, obtained from equilibrium thermal denaturation experiments.

The dependence of the activation free energy on temperature can be described following the transition state theory as[4]

$$\Delta G^{TS}(T) = \Delta H^{TS}(T_0) - T\Delta S^{TS}(T_0) + \Delta c_p^{TS}[(T - T_0) - T\ln\frac{T}{T_0}] \qquad \text{(Eq. S3)}$$

where ΔH^{TS} is the activation enthalpy, T is the absolute temperature in Kelvin, T_0 is a temperature of reference, ΔS^{TS} is the activation entropy and Δc_p^{TS} is the change in heat capacity. Then, by fitting Eyring's equation[4] to the folding and unfolding rate constants the following equations may be derived

$$k_F = \varepsilon \frac{k_B}{h} e^{[\Delta S_F^{TS}(T_0)]/R} T e^{\{ -\Delta H_F^{TS}(T_0) - \Delta c_{p,F}^{TS}[(T-T_0) - T \ln \frac{T}{T_0}] \}/RT} \qquad \text{(Eq. S4)}$$

and

$$k_U = \varepsilon \frac{k_B}{h} e^{[\Delta S_U^{TS}(T_0)]/R} T e^{\{ -\Delta H_U^{TS}(T_0) - \Delta c_{p,U}^{TS}[(T-T_0) - T \ln \frac{T}{T_0}] \}/RT} \qquad \text{(Eq. S5)}$$

where ε is the transmission coefficient, k_B is Boltzmann's constant, h is the Planck constant and ΔS_F^{TS}, ΔH_F^{TS}, $\Delta c_{p\,F}^{TS}$ are the change in entropy, enthalpy and heat capacity of folding respectively, while ΔS_U^{TS}, ΔH_U^{TS}, $\Delta c_{p\,U}^{TS}$ are the change in entropy, enthalpy and heat capacity of unfolding.

The observed relaxation rate constants, together with the deconvoluted folding and unfolding components, for wild type frataxin and its site directed mutants are reported in Figure S10. In order to ensure sample conductivity, all experiments (including equilibrium) were carried out in the presence of 50 mM sodium sulfate. The calculated changes in free energies for the transition and native states upon mutation and their associated Φ value are reported in Table S1. Because of the complexity of the analysis, we chose to focus our calculations at experimental conditions that could be directly explored by T-jump, avoiding extrapolation at higher or lower temperatures, and obtained for each mutant two sets of Φ values, referring to the transition states at high (323 K) and low (287 K) temperature. As detailed below, the experimentally determined Φ values were used as restrained in molecular dynamics simulations, to determine the ensemble structure of the hot and cold transition states for folding.

Thermal denaturation was followed on a JASCO circular dichroism (CD) spectropolarimeter (JASCO, Inc., Easton, MD), in a 1-mm quartz cuvette at 222 nm. Protein concentration was typically 10-20 μM. The relaxation kinetics were measured by using a Hi-Tech PTJ-64 capacitor-discharge T-jump apparatus (Hi-Tech, Salisbury, UK). Temperature was rapidly changed with discharge of about 35 kV on the solution, corresponding to a jump-size of 9 K.

Usually 10–20 individual traces were averaged. The fluorescence change of N-acetyltryptophanamide (NATA) was used in control measurements. Degassed and filtered samples were slowly pumped through the 0.5 x 2 mm quartz flow cell before data acquisition. The excitation wavelength was 296 nm and the fluorescence emission was measured using a 320 nm cut-off glass filter. Protein concentration was typically 10-20 μM. The buffer used in both equilibrium and kinetic experiments was 20 mM Hepes at pH 7.0 in the presence of 50 mM sodium sulfate and 2 mM DTT.

Supporting References

1 Marinelli, F., Pietrucci, F., Laio, A. & Piana, S. A kinetic model of trp-cage folding from multiple biased molecular dynamics simulations. *PLoS Comp. Biol.* **5**, e1000452, (2009).

2 Ceriotti, M., Tribello, G. A. & Parrinello, M. Simplifying the representation of complex free-energy landscapes using sketch-map. *Proc. Natl. Acad. Sci. USA* **108**, 13023-13028, (2011).

3 Fersht, A. R., Matouschek, A. & Serrano, L. The folding of an enzyme: I. Theory of protein engineering analysis of stability and pathway of protein folding. *J. Mol. Biol.* **224**, 771-782, (1992).

4 Fersht, A. R. *Structure and mechanism in protein science: A guide to enzyme catalysis and protein folding*. (W. H. Freeman, 1999).

5 Vendruscolo, M., Paci, E., Dobson, C. M. & Karplus, M. Three key residues form a critical contact network in a protein folding transition state. *Nature* **409**, 641-645, (2001).

6 Bonetti, D. *et al.* The kinetics of folding of frataxin. *Phys. Chem. Chem. Phys.* **16**, 6391-6397, (2014).

7 Fersht, A. R. & Sato, S. Φ-value analysis and the nature of protein-folding transition states. *Proc. Natl. Acad. Sci. USA* **101**, 7976-7981, (2004).

8 Gianni, S. *et al.* Understanding the frustration arising from the competition between function, misfolding, and aggregation in a globular protein. *Proc. Natl. Acad. Sci. USA* **111**, 14141-14146, (2014).

Table S1. Kinetic and equilibrium folding parameters of site directed mutants of frataxin under cold and hot conditions.

mutant	$\Delta\Delta G_{D\text{-}TS}$ (kcal mol^{-1})	$\Delta\Delta G_{D\text{-}N}$ (kcal mol^{-1})	Φ	$\Delta\Delta G_{D\text{-}TS}$ (kcal mol^{-1})	$\Delta\Delta G_{D\text{-}N}$ (kcal mol^{-1})	Φ
	**Cold denaturation pathway (287 K)**			**Hot denaturation pathway (323 K)**		
**L19A**	0.55 ± 0.05	0.57 ± 0.06	0.02 ± 0.02	0.62 ± 0.02	0.71 ± 0.07	0.13 ± 0.02
**Y22A**[b]	0.86 ± 0.08	1.3 ± 0.1	0.34 ± 0.04[b]	1.85 ± 0.17	1.1 ± 0.2	-0.72 ± 0.09[b]
**A26G**[b]	1.10 ± 0.09	1.8 ± 0.2	0.38 ± 0.07[b]	1.85 ± 0.15	1.30 ± 0.1	-0.42 ± 0.04[b]
**Y29A**	0.63 ± 0.07	1.6 ± 0.2	0.60 ± 0.09	1.01 ± 0.11	1.2 ± 0.1	0.15 ± 0.01
**L30A**[b]	0.27 ± 0.03	1.6 ± 0.2	0.83 ± 0.11[b]	1.49 ± 0.17	1.2 ± 0.2	-0.23 ± 0.03[b]
**L33A**[b]	-0.35 ± 0.02	0.9 ± 0.1	1.4 ± 0.2[b]	0.57 ± 0.03	1.0 ± 0.1	0.46 ± 0.06[b]
**L37A**	0.18 ± 0.02	1.4 ± 0.1	0.88 ± 0.09	1.24 ± 0.14	2.1 ± 0.2	0.40 ± 0.06
**V51A**	0.21 ± 0.03	0.78 ± 0.09	0.73 ± 0.08	0.93 ± 0.13	0.9 ± 0.1	0.02 ± 0.04
**L53A**	1.00 ± 0.15	2.1 ± 0.3	0.53 ± 0.09	1.09 ± 0.09	1.7 ± 0.2	0.36 ± 0.05
**L60A**	0.39 ± 0.06	1.2 ± 0.1	0.67 ± 0.07	0.02 ± 0.01	1.73 ± 0.2	1.0 ± 0.1
**I62V**[a]	-0.46 ± 0.06	-0.16 ± 0.04	-[a]	-0.13 ± 0.02	0.10 ± 0.04	- [a]
**A64G**[b]	0.53 ± 0.03	0.72 ± 0.06	0.27± 0.03[b]	1.42 ± 0.08	1.1 ± 0.1	-0.32 ± 0.04[b]
**I70V**[a]	-0.55 ± 0.07	0.09 ± 0.05	- [a]	0.10 ± 0.01	0.87 ± 0.07	0.88 ± 0.09
**L81A**[a]	2.1 ± 0.7	0.36 ± 0.02	- [a]	0.34 ± 0.1	0.87 ± 0.04	0.6 ± 0.2
**L107A**	0.63 ± 0.04	1.6 ± 0.2	0.61 ± 0.08	0.91 ± 0.06	1.1 ± 0.1	0.20 ± 0.03
**T108S**[a]	-0.09 ± 0.03	-0.02 ± 0.02	- [a]	0.08 ± 0.03	0.05 ± 0.02	- [a]
**V115A**	0.08 ± 0.05	1.1 ± 0.1	0.9 ± 0.1	0.51 ± 0.03	1.1 ± 0.1	0.54 ± 0.05
**A118G**	0.37 ± 0.02	1.3 ± 0.1	0.7 ± 0.1	0.81 ± 0.04	1.1 ± 0.1	0.29 ± 0.04
**I119V**	0.28 ± 0.02	1.3 ± 0.2	0.8 ± 0.1	0.42 ± 0.03	0.8 ± 0.1	0.5 ± 0.1

The mutants I68A, I79V and L111A expressed poorly and were denatured at the investigated experimental conditions.

[a]These mutants display thermodynamic stabilities too similar to wild-type frataxin (ΔΔGeq <0.4 kcal mol−1), which prevents accurate calculation of Φ-values [7].

[b]These mutants display non standard Φ-values (Φ-values higher than 1 or less than 0) and therefore were not used in restrained molecular dynamics simulations. The presence of unusual Φ-values in frataxin has been previously described, on the light of the high level of frustration of this protein [8].

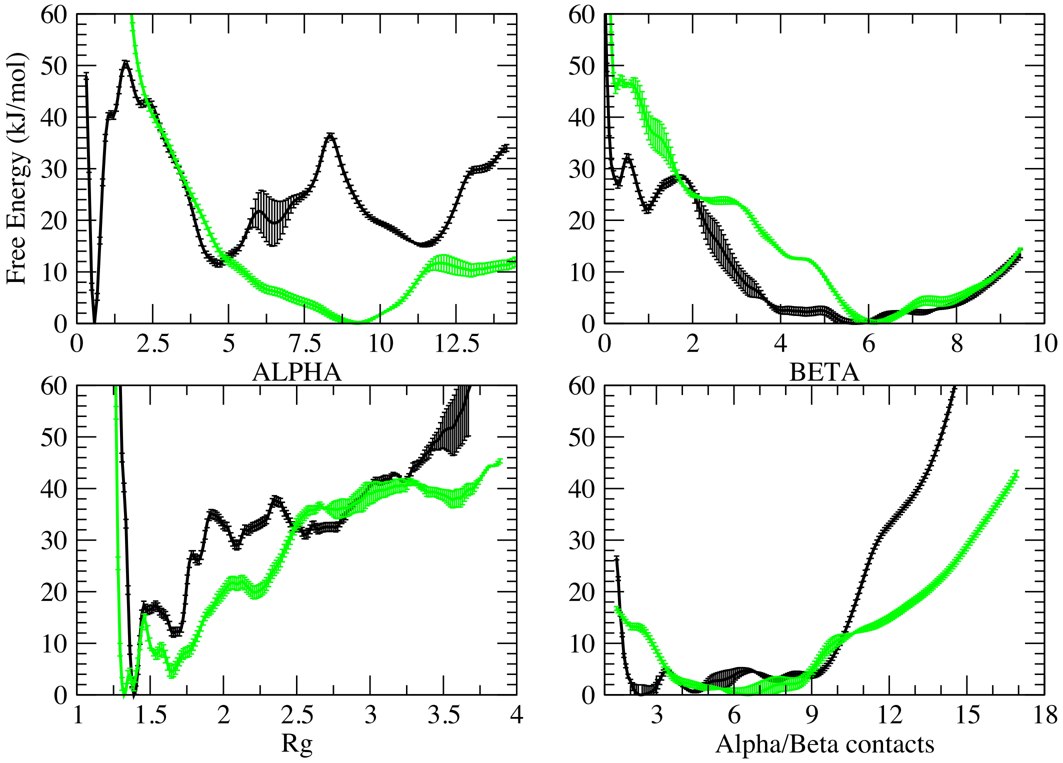

Figure S1. Convergence of the free-energy profiles along the four CVs used in the RAM simulations. Black profiles represent the CDS simulations and green ones the HDS simulations. The average errors in the free energy are 2.5 kJ/mol for the CDS and 1.8 kJ/mol for the HDS.

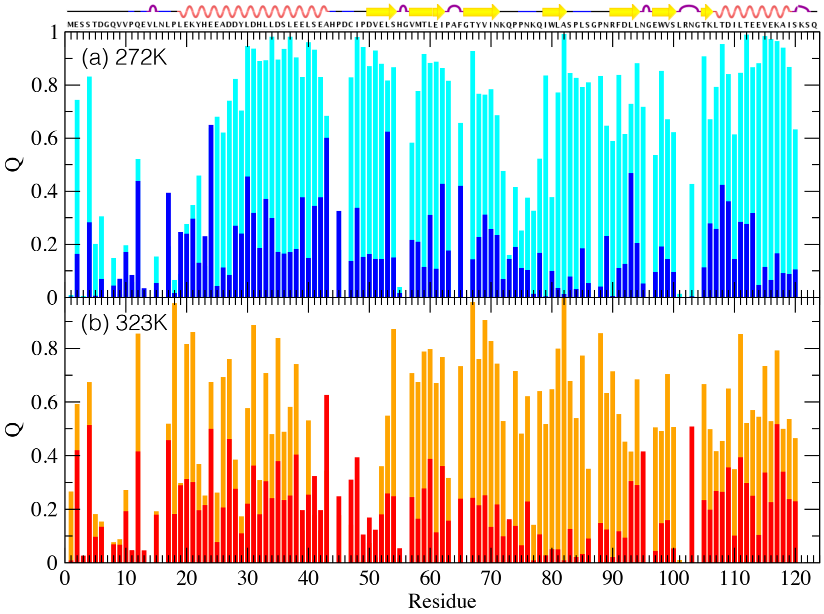

Figure S2. Fraction of native contacts (Q) per residue for the CDS and CTS (upper panel, blue and light blue, respectively) and for the HDS and HTS (lower panel, red and orange, respectively).

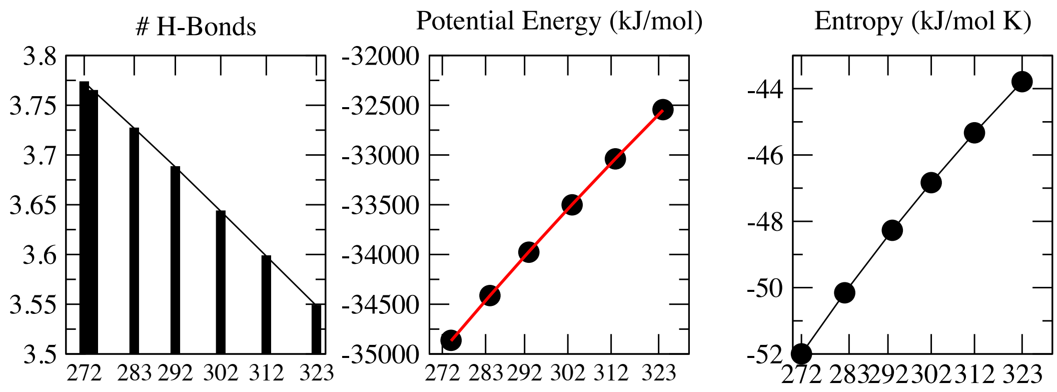

Figure S3. Properties of TIP4P05 water molecules for increasing temperatures, from 272 K to 323 K. From the left are reported the number of hydrogen bonds per molecule, the average potential energy and the entropy, which was calculated from:

$$S(T) = \frac{E(T)}{T} - \int_{T}^{+\infty} \frac{E(T')}{T^2} dT'$$

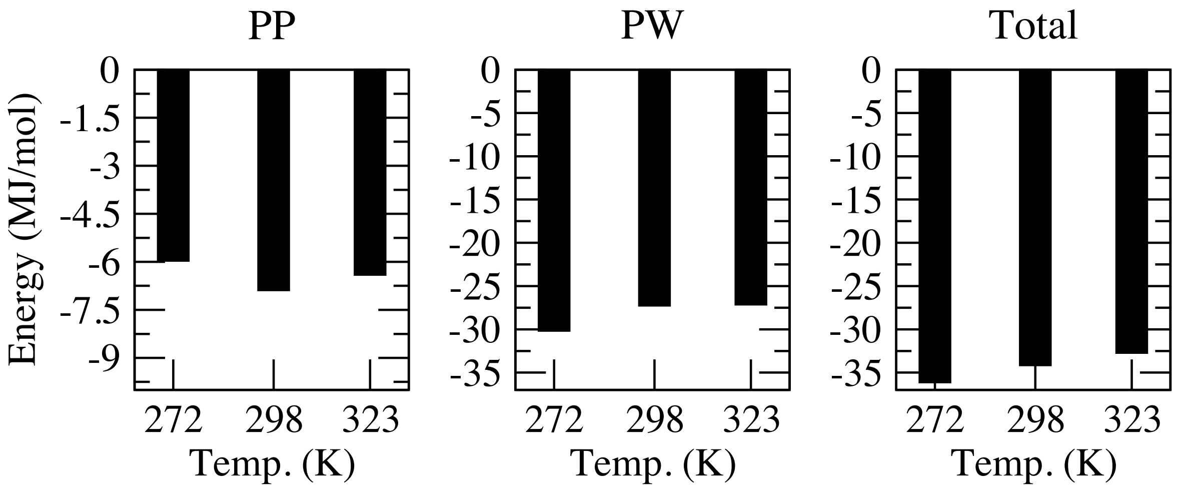

Figure S4. Protein-protein (PP), protein-water (PW) and total van der Waals and Coulomb energies for the CDS, NS and HDS ensembles of frataxin. While PP energy is minimized in the native state (298 K) the PW and the total interaction energies increase with the temperature.

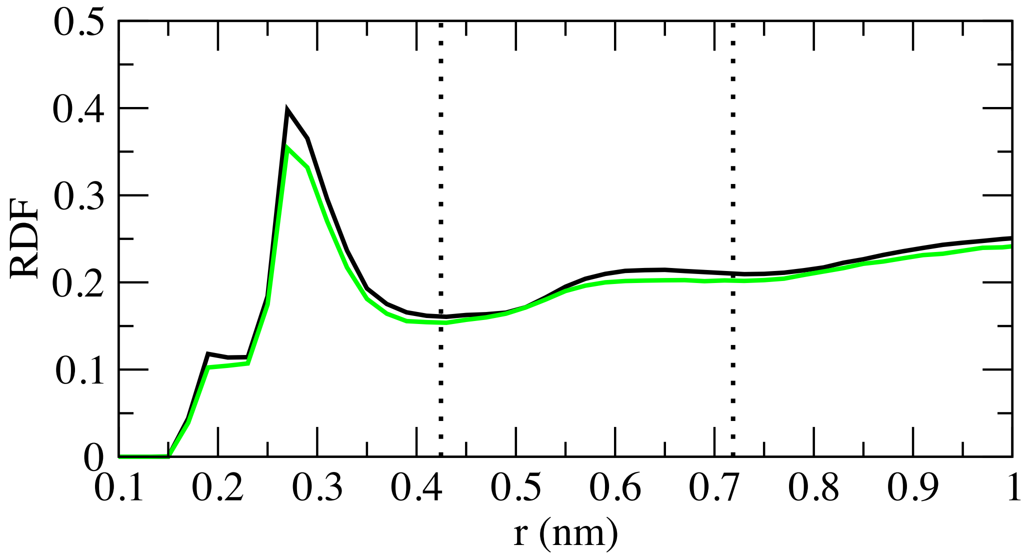

Figure S5. Radial distribution functions for the water in the CDS (black) and the HDS (green) with respect to the surface of the protein.

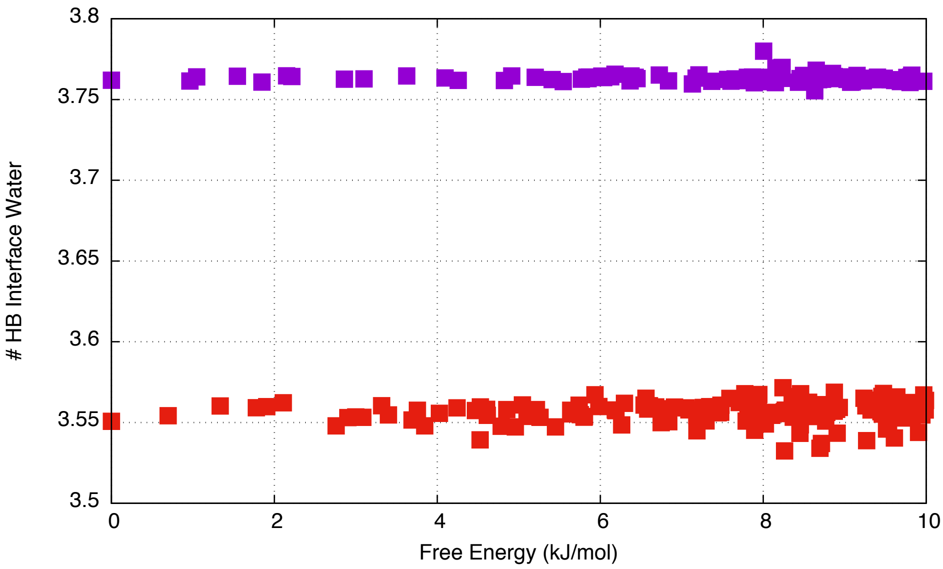

Figure S6. Both at low (purple) and high (red) temperatures the number of hydrogen bonds per interface water molecule does not depend on the free energy of the protein conformation.

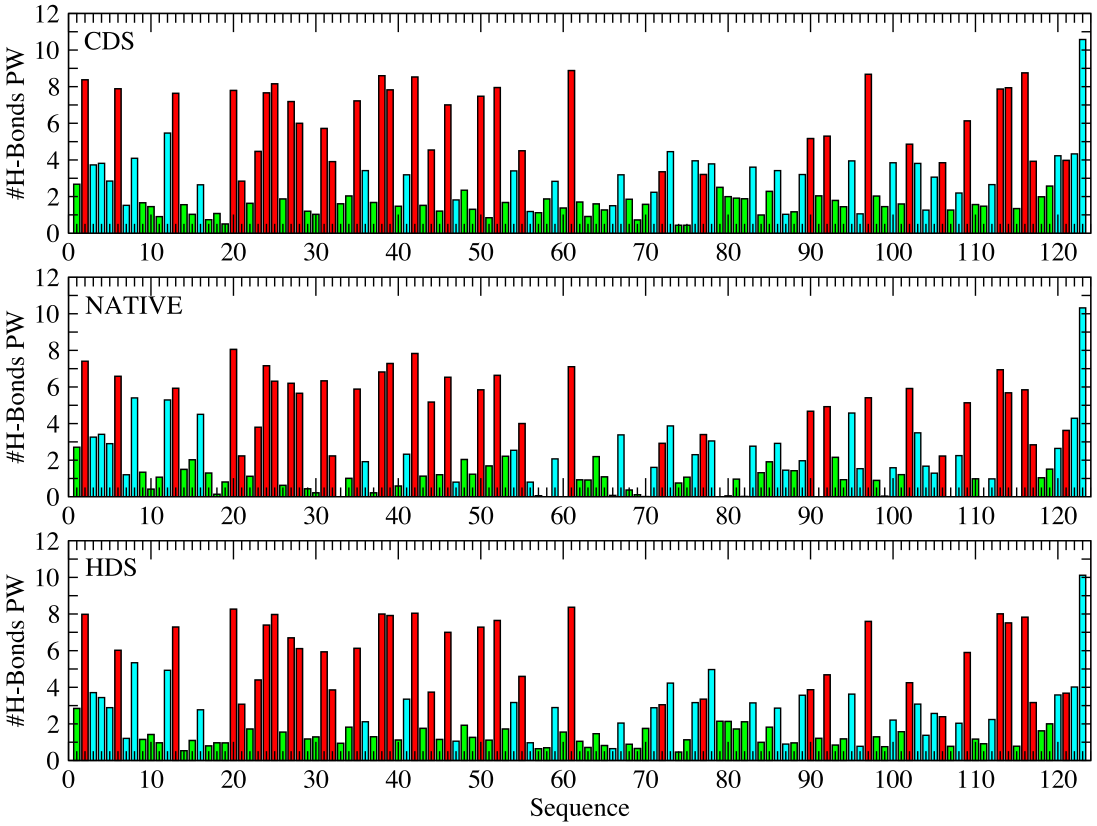

Figure S7. Number of Protein-Water (PW) hydrogen bonds per residue in the CDS, NS and HDS ensembles. The colour code is: cyan (polar), red (charged) and green (hydrophobic).

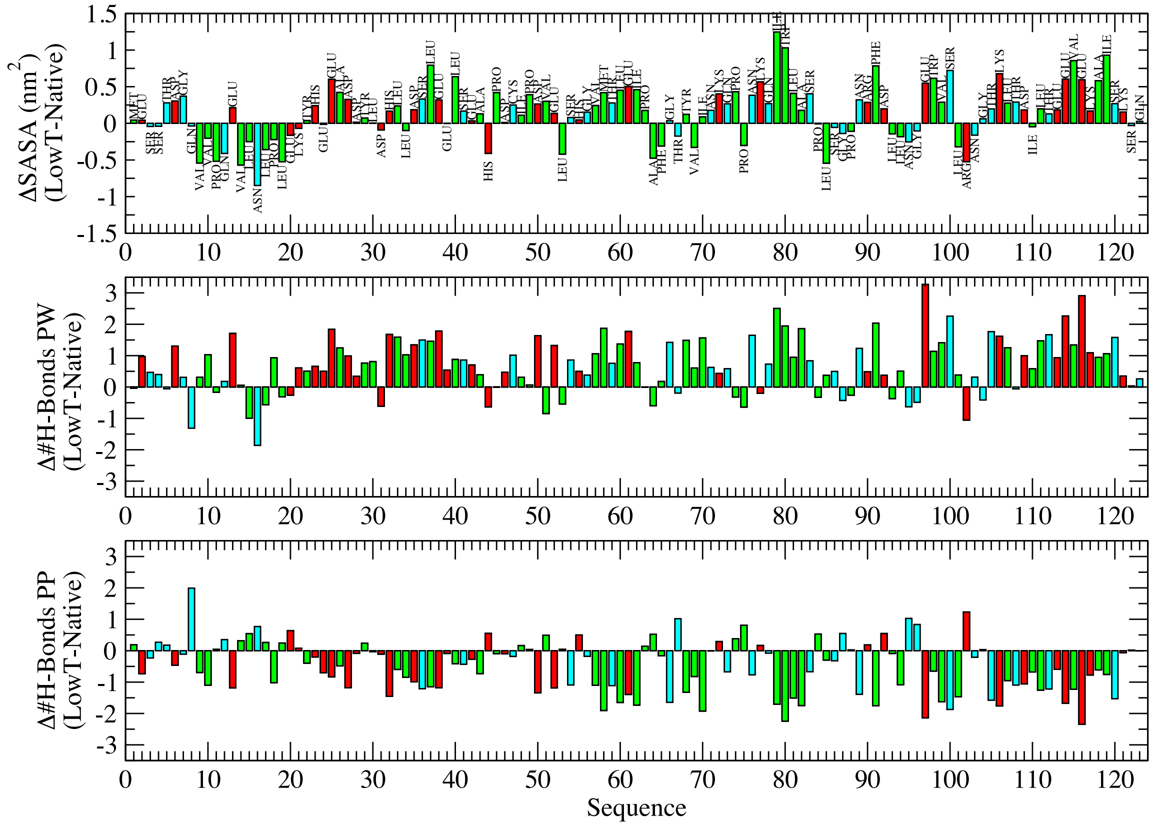

Figure S8. Per residue differences between the CDS and the NS ensembles for SASA, Protein-Water (PW) hydrogen bonds and Protein-Protein (PP) hydrogen bonds. The colour code is: cyan (polar), red (charged) and green (hydrophobic).

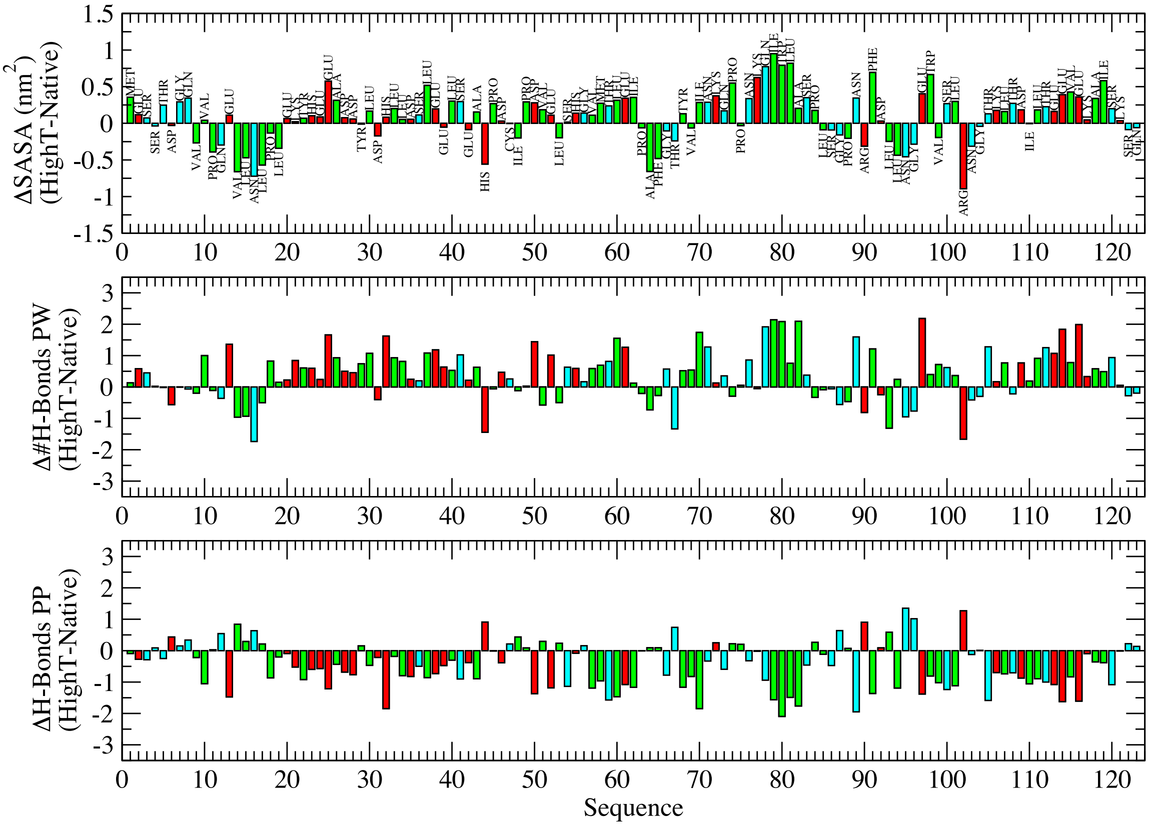

Figure S9. Per residue differences between the HDS and the NS ensembles for SASA, Protein-Water (PW) hydrogen bonds and Protein-Protein (PP) hydrogen bonds. The colour code is: cyan (polar), red (charged) and green (hydrophobic).

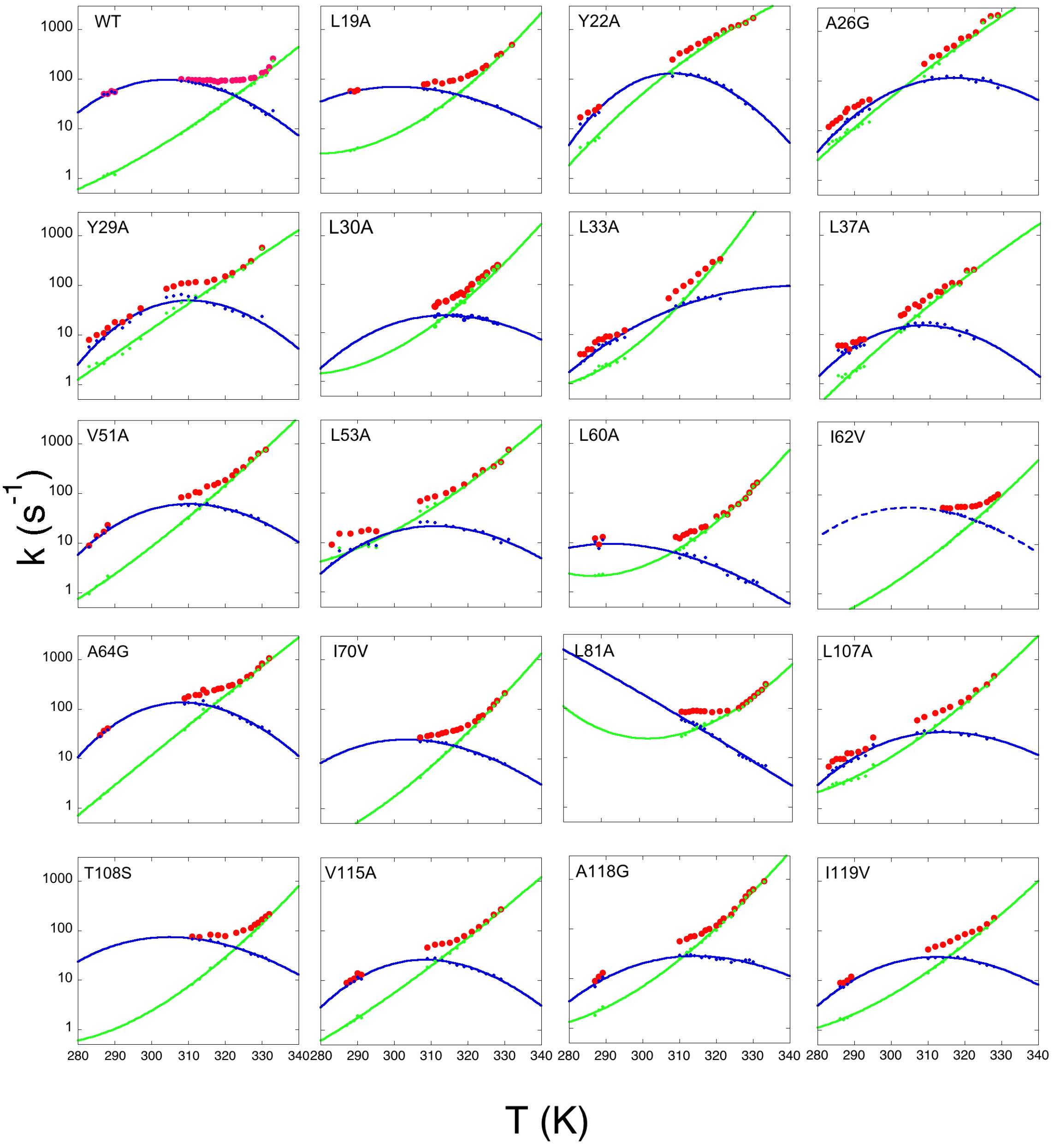

Figure S10. Kinetic and equilibrium folding parameters of site directed mutants of frataxin under hot and cold conditions (see also **Table S1**).

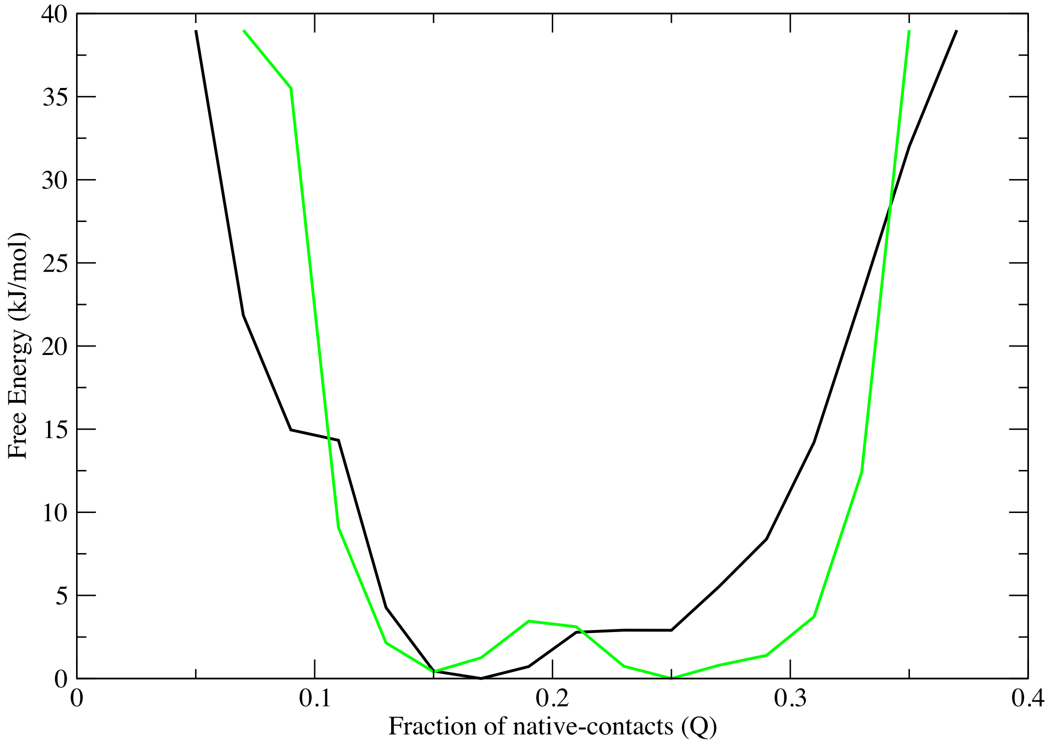

Figure S11. Free energy profiles for the CDS (black) and the HDS (green) as a function of the fraction of native-contacts (Q). The two profiles show that the HDS exhibits more native contacts than the CDS, but also that the free energy barrier for folding is steeper for the HDS than the CDS.
